# Breast schwannoma in a patient with prior breast cancer: a rare case report

**DOI:** 10.1097/RC9.0000000000000719

**Published:** 2026-07-28

**Authors:** Kem-Maria McCook, Nisseau-Bey Zhuri, Chevar South, Zarrin Hosseinzadeh, Theodora Abah, Joel Okoli

**Affiliations:** a Department of Surgery, Morehouse School of Medicine, Atlanta, GA, USA; b Department of Pathology and Laboratory Medicine, Emory University School of Medicine, Atlanta, GA, USA

**Keywords:** breast, breast cancer, case report, peripheral nerve sheath tumors, schwannoma

## Abstract

**Introduction:**

Breast schwannoma is a very rare benign peripheral nerve sheath tumor, accounting for approximately 0.2% of all breast tumors. With fewer than 70 cases reported in the literature, the diagnosis is usually challenging and is often not considered.

**Case presentation:**

We present a case of a 62-year-old Black female with a history of right breast cancer, status post right modified radical mastectomy, and chemoradiation 30 years ago, without prior evidence of recurrence. She presented with a mass in the left breast. Initial diagnostic mammogram and ultrasound reported the mass as a benign lymph node. A repeat mammogram showed interval growth, prompting an ultrasound-guided core-needle biopsy. Histological results were consistent with schwannoma. The patient underwent a left I-125 radioactive seed-localized partial mastectomy for definitive diagnosis and treatment, due to her high risk of breast cancer and preference.

**Discussion:**

This report highlights the importance of continued clinical and radiographic surveillance in patients with a history of breast cancer, as new breast lesions may warrant further evaluation despite benign-appearing imaging characteristics. Although rare, breast schwannoma should be included in the differential diagnosis of benign breast masses. While breast schwannomas may be managed with observation, in select cases, surgical excision provides a definitive histopathologic diagnosis and is curative when intervention is clinically indicated.

**Conclusion:**

A low threshold for tissue diagnosis is warranted in breast cancer survivors who present with new breast lesions, even when imaging characteristics appear benign. Management of benign-appearing breast masses should be individualized, and surgical excision for definitive diagnosis and treatment should remain a consideration in cases of interval growth, persistent concern for occult malignancy, or when ongoing imaging surveillance poses a significant burden or inconvenience to the patient.

## Introduction

Breast lumps or masses are widespread, particularly those due to fibrocystic changes of the breast and fibroadenomas. Breast schwannomas, however, are exceptionally rare, with approximately 60–65 cases documented in the literature to date, and recent cumulative reviews suggesting at least 55 previously reported cases before the addition of more recent reports[1]. Table 1 summarizes previously reported cases and reviews of breast schwannomas in the literature, underscoring the rarity of this entity and the limited cumulative clinical experience described to date.

**Table 1:** Comparative summary of reported breast schwannoma cases in the literature.

Study	Year	Study type/number of cases	Key findings
Collins & Gau[2]	1973	First reported case	First documented case of breast neurilemmoma
Gupta *et a*l[3]	2000	Two-case report (male and female patients)	Demonstrated occurrence in both sexes
Balci *et al*[4]	2009	Imaging-focused case report	Described benign mammographic and ultrasonographic appearance
Halteh *et al*[5]	2017	Single case report with literature review	Reinforced rarity of breast schwannoma (~0.2% of breast tumors)
Gorji *et al*[6]	2021	Literature review	Identified approximately 25 previously reported English-language cases
Jain *et al*[7]	2023	Literature review	Reported approximately 55 cumulative published cases
Li *et al*[1]	2025	Two-case report and literature review	Added contemporary cases and reaffirmed benign imaging and histopathologic characteristics

Breast schwannomas can occur across all age groups, although they are most commonly observed in individuals between 20 and 50 years of age [8]. Collins *et al* were the first to report breast schwannomas in 1973[2]. Das Gupta *et al* reported that breast schwannomas accounted for 2.6% of all reported schwannomas, corroborating their rarity compared with other anatomical sites[9].

Within the limited literature available, the incidence of breast schwannoma in men and women is fairly equal, with documented cases in both sexes[3]. More commonly, breast tumors tend to occur in the upper outer quadrant, but schwannomas do not show a similar predilection and can affect all quadrants simultaneously[9]. They represent approximately 0.2% of all breast tumors[5]. Recent literature reviews further demonstrate that these tumors may arise in unusual locations, including the nipple-areolar complex, and may clinically mimic fibroadenomas or other benign breast lesions^[^1,6^]^. Due to their low incidence in the breast, there are limited reports regarding the ultrasound and mammographic findings of these tumors. The present case is particularly relevant because it describes the occurrence of a benign breast schwannoma in a patient with a prior history of contralateral breast carcinoma. This clinical scenario can significantly heighten suspicion for recurrent or metastatic disease. In this context, the case highlights the importance of maintaining a broad differential diagnosis for newly identified breast masses, even in patients with a known oncologic history.

HIGHLIGHTS

- A breast schwannoma is an extremely rare benign peripheral nerve sheath tumor.
- Fewer than 70 cases are reported in the literature.
- Imaging may mimic benign lesions such as lymph nodes.
- Definitive diagnosis requires histopathologic confirmation.
- Surgical excision is curative with a favorable prognosis.

Balci *et al* evaluated the classic mammographic and ultrasonographic appearance of schwannomas, which typically present with features resembling those of benign tumors. They determined that the clarity and shape of the tumor capsule, as well as the internal hypoechoic characteristics on ultrasound, were key to differentiating breast schwannomas from other benign breast masses. High-frequency mammography, when combined with ultrasound, further increased the diagnostic accuracy[4]. More recent reports have similarly demonstrated that breast schwannomas most commonly appear as well-circumscribed, oval or round hypoechoic lesions on ultrasonography and are frequently categorized as Breast Imaging Reporting and Data System (BI-RADS) 3 or 4A lesions because of their indeterminate but predominantly benign imaging characteristics^[^6,7^]^. Notably, axillary lymph node enlargement is typically absent in all reported cases.

Like any other tumor, the diagnosis of schwannoma relies on histopathological examination. Tissue evaluation usually reveals the classic features of a schwannoma, indistinguishable from schwannomas at any other anatomical site, as described by Fujii *et al*[10]. Histologically, these tumors characteristically demonstrate Antoni A and Antoni B areas with Verocay body formation and diffuse S100 protein positivity on immunohistochemical staining, findings that remain essential for definitive diagnosis^[^1,6,7^]^.

## Case presentation

This is a case of a 62-year-old African American female with a history of right breast cancer, status post right modified radical mastectomy followed by chemoradiation therapy in 1992, who has had no evidence of recurrent disease. Her annual left screening mammograms were read as normal, BI-RADS category 1, until March 2023, when she palpated a painless left breast mass. She has no personal or family history of neurofibromatosis. On physical examination, a mobile, firm, non-tender 1 cm mass was noted at 11 o’clock, non-adherent to the overlying skin or underlying tissue. She underwent a diagnostic mammogram that showed a mass in the upper inner quadrant of the left breast, correlating with the palpable abnormality at 11 o’clock. Targeted ultrasound characterized the mass as a benign-appearing lymph node at the 11 o’clock position, 23 cm from the nipple, BI-RADS category 2. Consistent with recommendations for BI-RADS category 2 findings, a core needle biopsy was not indicated, and the patient was advised to continue routine annual screening; this plan remained unchanged in 2024.

**Figure 1.**
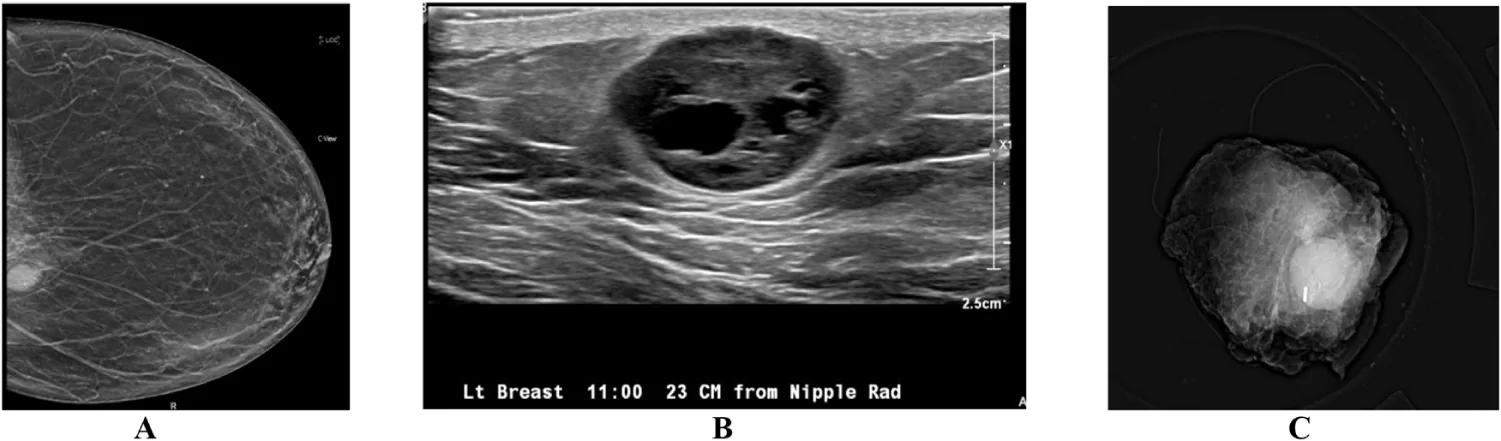
(A) Mammogram showing a well-defined radiopaque mass in the left breast. (B) Ultrasound showing a 1.9 × 1.7 × 1.4 cm oval complex solid and cystic mass with mixed hypoechoic and hyperechoic areas. (C) Specimen radiograph showing excised mass, radioactive seed, and biopsy clip.

Routine left screening mammogram in 2025 showed a mass in the exact location previously characterized as a lymph node that had increased in size from 1.0 to 2.0 cm and was assigned BI-RADS category 0, as shown in Figure 1A. Subsequent ultrasound showed an oval, complex, solid-and-cystic mass with mixed hypo- and hyperechoic tissue measuring 1.9 × 1.7 × 1.4 cm (BI-RADS category 4A; Fig. 1B). She underwent ultrasound-guided core-needle biopsy on 19 May 2025, with the pathology showing a bland, S100 +, spindle-cell neoplasm with hypercellular and hypocellular areas, consistent with a schwannoma. Genetic testing showed no pathogenic variants. After a multidisciplinary discussion, she was presented with the options of observation versus surgical excision. She requested a second opinion and was therefore referred to our clinic for this purpose. We discussed the options of clinico-radiologic surveillance versus excision in great detail and expressed our concern regarding the significant increase in the size of the tumor. She opted for immediate excision of the tumor for histopathologic evaluation of the entire mass, in the rare event of a false-negative core-needle biopsy. She wanted the peace of mind of no longer having the mass.

**Figure 2.**
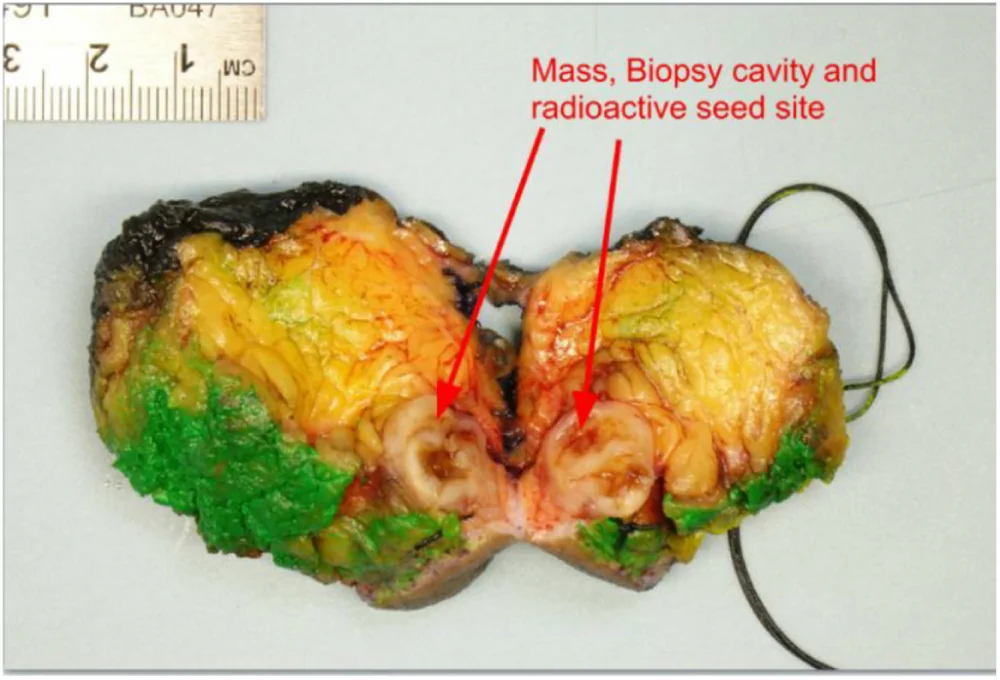
Tan-yellow and lobulated adipose tissue with a well-circumscribed tan-white 2.3 cm mass. The cut surface of the mass appears solid to focally cystic and homogeneous.

**Figure 3.**
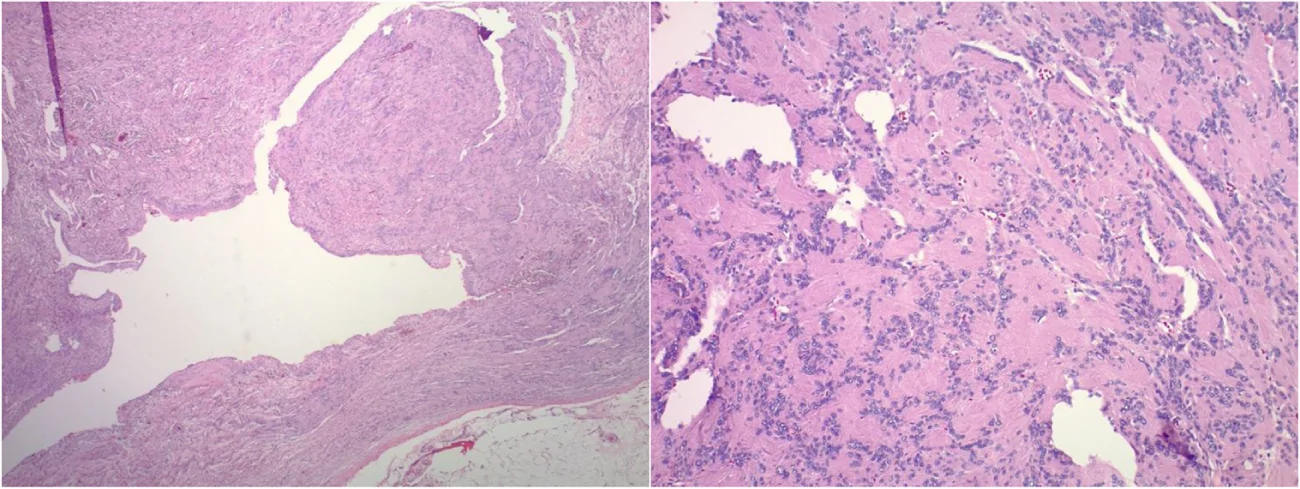
An encapsulated, bland spindle cell lesion with hypercellular (Antoni A) and hypocellular (Antoni B) regions. The cells have moderately eosinophilic cytoplasm with indistinct cell borders, inconspicuous nucleoli, and low mitotic activity.

On 8 August 2025, she underwent a left I-125 radioactive seed-localized partial mastectomy with complete excision of the tumor, including a rim of normal-appearing breast tissue and an attached overlying skin ellipse. An elliptical segment of skin containing the biopsy-site indentation was excised en bloc with the entire tumor. The procedure lasted 1 hour with 10 ml estimated blood loss. A Faxitron specimen radiograph confirmed the presence of the mass, biopsy clip, and radioactive seed (Fig. 1C). The final pathology results confirmed a 2.3 cm schwannoma with clear margins and a benign skin ellipse measuring 4 × 2 cm, located 0.4 cm from the mass. Figure 2 shows the gross pathology specimen. Histology confirmed a spindle cell lesion with hypercellular (Antoni A) and hypocellular (Antoni B) regions, consistent with a schwannoma, as shown in Figure 3.

She was discharged home on the day of surgery and had an uneventful post-operative course. She was seen in the surgical oncology clinic at 2 weeks, 3 months, and 6 months post-operatively, and she was recovering well with no clinical evidence of recurrence. She missed her annual screening mammogram in March 2026 and rescheduled it for 30 July 2026, when she will be due for a clinical examination. If her annual screening mammogram is normal, she will continue with routine annual screening mammograms.

## Discussion

Breast schwannomas are rare and make up only 2.6% of all schwannomas and approximately 0.2% of all breast tumors^[^5,9^]^. Our case was an apt demonstration of some of the challenges documented in the sparse literature on breast schwannomas. Breast schwannomas often pose a diagnostic challenge, as seen in our case, given their presentation with clinical and radiographic features similar to benign lesions such as lymph nodes and fibroadenomas. Normal mammographic findings have been reported previously in breast schwannomas, further highlighting the diagnostic enigma that breast schwannomas can pose^[^10,11^]^. Our patient’s second diagnostic ultrasound showed hypoechoic areas, which have been reported previously as a characteristic feature differentiating schwannomas from other benign lesions, though this finding is non-specific[4]. The benign differential diagnosis at this time included an intramammary lymph node, fibroadenoma, intraductal papilloma, phyllodes tumor, and schwannoma. Malignant considerations included metachronous contralateral breast cancer, given the patient’s history.

Given the benign biopsy findings, observation with interval clinico-radiologic surveillance every 6 months for 2 years (with a return to routine annual screening if no significant changes were observed) was discussed as a reasonable management option. However, surgical excision was also strongly considered, given several factors, including the patient’s prior history of contralateral breast cancer; interval doubling in lesion size over approximately 1 year; concerns regarding long-term compliance with imaging surveillance; the small but recognized risk of malignant peripheral nerve sheath tumor transformation reported in the literature; and the patient’s concern about having a persistent mass and decision to have it excised.

Benign peripheral nerve sheath tumors can rarely transform into malignant peripheral nerve sheath tumors (MPNSTs), especially in individuals with neurofibromatosis[12]. Schwannoma, a type of benign peripheral nerve sheath tumor, can rarely become a malignant soft tissue sarcoma; however, the incidence of malignant transformation in breast schwannomas is unknown. Definitive diagnosis and treatment of breast schwannoma, with excision, are reasonable in high-risk patients with an enlarging breast mass, as in our patient.

Although the lesion was clinically palpable, preoperative I-125 radioactive seed localization was utilized because the mass was small, mobile, and located superficially within the upper inner quadrant of the breast near the skin. Seed localization allowed for precise intraoperative targeting and facilitated accurate excision of the biopsy-proven lesion while minimizing removal of excess surrounding breast tissue. In addition, the patient’s prior history of contralateral breast cancer and interval enlargement of the lesion raised concern for possible discordance between imaging and core biopsy findings, making definitive localization of the biopsied lesion important.

A partial mastectomy approach, rather than a simple palpation-guided excisional biopsy, was selected to ensure complete excision of the lesion with a surrounding rim of grossly normal tissue and overlying skin, because of the lesion’s superficial location and continued concern for possible occult malignancy despite benign core biopsy pathology. Thus, the procedure was performed not only for therapeutic excision of an enlarging mass, but also as an oncologic precaution to obtain definitive histopathologic evaluation and to ensure a complete, margin-negative resection in a patient with a history of breast cancer. Although excision of an overlying skin ellipse is not routinely required, the lesion was located in close proximity to the skin (0.4 cm), with indentation of the skin at the biopsy site, necessitating excision of a skin ellipse around the biopsy site en bloc with the excised tumor.

Given that breast schwannoma is even rarer in older individuals and in patients with a personal history of breast cancer, a very high index of suspicion was warranted in our case to rule out a malignant lesion. Definitive diagnosis and treatment are achieved with surgical excision and histopathologic examination that demonstrates a spindle-cell lesion with hypercellular (Antoni A) and hypocellular (Antoni B) regions.

Given our patient’s histologic findings of the resected tumor, we can now definitively say she had a breast schwannoma that was mistakenly categorized as a benign lymph node. This case highlights the importance of radiologic surveillance in patients with a history of breast cancer and of tissue diagnosis of an evolving breast mass, even if benign in appearance. She effectively had a diagnostic and therapeutic operation, curing her of this condition. She can now return to routine annual breast cancer screening.

While schwannomas generally have a good prognosis, there are no data on the rate of recurrence of breast schwannomas. The surveillance of this patient should follow the general principles of benign breast tumors. It is of utmost importance that she continues to undergo radiologic surveillance with mammograms, given her personal history of breast cancer and the presence of this benign peripheral nerve sheath tumor.

## Conclusion

A low threshold for tissue diagnosis is warranted in breast cancer survivors who present with new breast lesions, even when imaging characteristics appear benign. Management of benign-appearing breast masses should be individualized, and surgical excision for definitive diagnosis and treatment should remain under consideration in cases of interval growth or persistent concern for occult malignancy, or when ongoing imaging surveillance poses a significant burden or inconvenience to the patient.

## Ethical approval

Per our institution’s policy, ethics approval is not required for case reports. As such an IRB exemption was obtained from our institution, Morehouse School of Medicine.

## Consent

Written informed consent was obtained from the patient for publication of this case report and accompanying images. A copy of the written consent is available for review by the Editor-in-Chief of this journal on request.

## Data Availability

The data supporting the findings of this study are included in the article. Further details are not publicly available due to patient confidentiality.
